# Pathological gynecomastia in children at Cipto Mangunkusumo hospital Jakarta

**DOI:** 10.1186/1687-9856-2013-S1-P64

**Published:** 2013-10-03

**Authors:** Novina Andriana, Frida Soesanti, Aman B Pulungan

**Affiliations:** 1 Division of Pediatric Endocrinology, Department of Pediatrics, Faculty of Medicine, Padjadjaran University, Bandung, Indonesia; 2 Division of Pediatric Endocrinology, Department of Pediatrics, Faculty of Medicine, University of Indonesia, Jakarta, Indonesia

## 

Gynecomastia is generally attributed to conditions that disrupt sex-steroid signaling pathways, resulting in increased or unopposed estrogen action on breast tissue. Pubertal gynecomastia is common and usually physiological, with sympathetic reassurance and watchful waiting the mainstays of treatment. Meanwhile, pathological gynecomastia should be identified, sought the cause and gave proper management shortly. The aim of our study was to raise vigilance into pathological gynecomastia that require more complex management and have long-term effect. We conducted a retrospective chart review of 12 patients with gynecomastia who presented to pediatric endocrinology clinic at Cipto Mangunkusumo Hospital, Jakarta from September 2009 to June 2012. Seven patients (58 %) aged 10.3-to 13.5 yr old were diagnosed physiologic pubertal gynecomastia, one patient (8 %) aged 1 month old was diagnosed neonatal gynecomastia and the rest 4 patients (34 %) were pathologic. Of the pathologic case, two patients aged 7.4 and 8.4 yr old were diagnosed prepubertal gynecomastia with history of taking herbal medicine and fast foods at least three times a week and still being observed. The others, aged 10.6 and 14.9 yr old were investigated and confirmed to have DSD (disordered of sexual development) 47 XXY, Klinefelter syndrome and DSD 46 XX ovotesticular. Our results suggested that gynecomastia in children should prompt an immediate evaluation distinguish a normal developmental variant from possible endocrine disorder in order to give the best treatment.

